# Effects of weight management program on postural stability and neuromuscular function among obese children: study protocol for a randomized controlled trial

**DOI:** 10.1186/s13063-015-0673-6

**Published:** 2015-04-10

**Authors:** Fenghua Sun, Li-Juan Wang, Lin Wang

**Affiliations:** Department of Health and Physical Education, The Hong Kong Institute of Education, Hong Kong, China; School of Sport Leisure, Recreation and Arts, Shanghai University of Sport, Shanghai, China; School of Kinesiology, Shanghai University of Sport, Shanghai, China

**Keywords:** Childhood obesity, Neuromuscular function, Postural stability, Randomized controlled trial, Weight loss

## Abstract

**Background:**

Childhood obesity is one of the most critical public health problems in the world. It is associated with low neuromuscular function and postural deformities. Whether weight loss can improve postural stability and neuromuscular control, benefit daily activities, or prevent injury is unknown. Therefore, this study attempts to investigate the effect of a 6 month weight management program on postural stability and neuromuscular control among obese children.

**Methods/design:**

We will conduct a prospective, single-blind, randomized controlled trial with 120 prepubescent obese children. Participants will be randomly assigned to a weight management group or a control group. The weight management group will participate in a dietary and exercise program. The control group will receive health education. After the intervention, participants will be followed for 6 months with no active intervention. The primary and secondary outcomes will be assessed at the baseline, and after 6 months and 12 months. Primary outcome measures will include body weight, body height, body mass index, waist circumference, hip circumference, and body fat percentage. Secondary outcome measures will include three-dimensional functional biomechanics in different tasks, proprioception tests of the knee and ankle, neuromuscular response of the leg muscles, and muscle strength tests of the knee and ankle. Furthermore, adverse events will be recorded and analyzed. An intention-to-treat analysis will be performed if any participants withdraw from the trial.

**Discussion:**

The important features of this trial include the randomization procedures and large sample size. This study attempts to estimate the effect of weight loss intervention on outcomes, including daily life function, postural stability, and neuromuscular control in prepubescent obese children. Therefore, our results can be useful for obese children, medical staff, and healthcare decision makers.

**Trial registration:**

Chinese Clinical Trial Registry ChiCTR-IOB-15005874.

## Background

Childhood obesity is one of the most critical public health challenges of the twenty-first century [1]. Evidence shows that childhood and adolescent obesity are significantly associated with the presence and clustering of cardiovascular risk factors in childhood [2]. Other childhood clinical consequences include asthma, Type 1 diabetes, low-grade systemic inflammation, sleep apnea, and musculoskeletal disorders, particularly of the lower limbs and feet [2,3]. Childhood obesity is also associated with a range of functional problems, including pain, discomfort and joint stiffness (particularly in the lower extremities), lower muscular strength, and postural deformities [4].

Childhood is the most important stage in growth and development. Being overweight or obese during this period can influence movement ability and postural control capacity [4]. Several studies reported the difficulties encountered by overweight and obese people when executing simple activities in daily living, particularly during weight-bearing tasks such as walking and climbing stairs [5,6]. These results suggest a possible link between movement efficiency in the gait cycle and decreased postural stability of obese children [7-11]. Several studies on the postural control of obese children published evidence of alterations in their postural control ability. These studies focused on the postural stability in obese children when walking or standing in different postures. Obese children displayed a longer cycle duration, lower cadence, lower velocity, and longer stance period than normal-weight subjects during walking [7-10]. Obese boys spent a significantly larger percentage of time in the double-support and stance phase than their non-obese counterparts during walking [7,11]. Furthermore, Deforche and colleagues [10] found that obese boys had slower weight transfer, lower rising index, and larger sway velocity in a sit-to-stand test than non-obese boys. The characteristics of the gait pattern indicate a decrease in dynamic postural stability in obese children. The static postural stability in obese children also decreased [9-11]. Previous studies show that static postural stability is lower in obese children than in normal-weight children. McGraw *et al.* [11] examined the sway of center of pressure during standing in obese boys in different vision conditions (that is, full vision, dark, and visual conflict). They found that the maximum displacement of center of pressure and the root mean square of center of pressure displacement in the anterior-posterior and medial-lateral directions tended to be larger for obese boys than for non-obese boys across all conditions. The difference in the measurements in the medial-lateral direction between obese and normal-weight children was most significant. Furthermore, a recent study suggests that obese and normal-weight children use different postural strategies to maintain balance, the consequences of which should be further clarified [12].

Obese children perform poorly in static and dynamic postural control [5]. The larger body size, larger body mass index, abnormal body fat distribution, and potential neuromuscular disadvantage of obese children can contribute to their postural instability [13]. Several studies of obese children provided evidence of this association between body weight or body shape and postural stability [11,14]. Furthermore, postural control is related to the function of vision, vestibular, proprioception, and neuromuscular control. Several studies previously demonstrated that information from some sensory receptors on obese individuals could be associated with postural instability [11,15]. McGraw *et al.* [11] found that visual conflict induced a more significant alternation of static postural performance in obese children than in their normal-weight counterparts. Several researchers reported a reduction in the sensitivity of plantar mechanoreceptors in obese feet [16]. This alteration might contribute to the postural instability in the obese group [4]. Obese children show a deficit in proprioception in knee flexion. Such alterations in their proprioception might be associated with decreased postural control in obese children [15]. Moreover, obese children have less relative strength during isokinetic testing [17]; the majority of deficits are evident where strength is a major prerequisite of functional movement tasks. Specifically, obese children typically perform poorly in measures of muscular strength that require moving the body against gravity [18,19]. Abdominal strength and endurance, functional knee strength, and lower extremity strength, power, and endurance are all low in this population [4].

Weight reduction for obese children is associated with decreased body mass index and body fat, increased physical fitness, and improved movement skills [20,21]. Previous studies found that weight loss in obese adults could improve their postural stability [22,23]. Obese children have worse postural stability and neuromuscular control than normal-weight children. However, only a few studies have examined the influence of weight reduction on postural balance among obese children. Recently, Steinberg *et al.* [24] found that a weight management program could improve static stability and lower potential falling probability of obese participants. However, it is not known whether weight loss interventions influence performance associated with dynamic postural instability and neuromuscular function in obese children.

Maintaining a stable posture is essential for many daily activities and injury prevention. The poor postural stability of obese children might lead to a higher risk of falling and fractures compared with that of normal-weight children [25,26]. In addition, childhood is the most important stage of human growth and development. If weight loss increases postural stability and neuromuscular control at this stage, it will benefit daily activities and help to prevent injuries. Therefore, this study attempts to investigate the effect of a 6 month weight management program on postural stability and neuromuscular control among obese children.

## Methods/design

### Study design

The study design is a single-blind randomized controlled trial that will compare a weight management program with health education. This study will be conducted at the Sport Medicine and Rehabilitation Center, Shanghai University of Sport. A total of 120 prepubescent obese children aged 8 to 10 years old will be included in the study. All participants will be recruited from a local primary school in Yangpu District, Shanghai, China. A multidisciplinary team composed of clinicians, dietitians, and exercise specialists will run the program.

Prior to initiation of the study, all subjects will complete a questionnaire that asks for details, such as medical history. The subjects will also complete the Mini-Mental State examination and Activity of Daily Living test, and describe their exercise habits (frequency and duration). Informed consent will be requested from all subjects and their parents prior to their inclusion in this study.

Participants who meet the inclusion criteria will be allocated in a 1:1 ratio by computer-generated randomization. After randomization, participants will be assigned to either the weight loss group or a health education group (control). This study will include assessments at the following time points: before intervention, after an intervention period of 6 months, and after a further follow-up period of 6 months with no active intervention. The total study period will be 12 months.

### Participants

#### Inclusion criteria

Participants with the following conditions will be included:

1. Obesity according to the childhood obesity definition of the International Obesity Task Force [27];
2. Age: 8 to 10 years;
3. Tanner stage 1 [28];
4. Availability: can participate in three exercise sessions per week for a period of 6 months;
5. At least one parent is willing to attend treatment meetings; and
6. No family member is currently involved in another weight control program.

#### Exclusion criteria

Participants with any of the following conditions will be excluded:

1. Inability to communicate in Chinese;
2. Diabetes or a psychiatric disorder (for example, schizophrenia, severe autism, mental retardation, or psychosis);
3. Exercise-induced or uncontrolled angina in the past 3 months or severe dyspnea at rest;
4. Medical condition that involves a syndrome cause of obesity or use of medication that influences weight gain or loss;
5. Other illnesses that render participation in this study inadvisable as judged by the participant or study physician.

#### Withdrawal criteria and management

Participants will be allowed or asked to withdraw from the study based on the following:

1. The participant or the participant’s legal representative makes such a request.
2. The participant develops a serious disease, such as heart disease or stroke, and continuing participation becomes unsuitable in the investigator’s opinion.
3. The participant has an adverse reaction related to the weight management program.

#### Participant recruitment

Participants will be recruited through advertisements in local newspapers and television, and from primary schools. Study details will be provided to the school administration, school teachers, and parents. Parents will be sent an invitation letter with a tear-off consent slip and a fact sheet. Participants and their parents who show interest in the weight management program will be included in this study.

#### Ethical considerations

This study was approved by the ethics committee of the Shanghai University of Sport (Reference number, 2014–12). Informed consent will be obtained from all participants their parents.

### Interventions

#### Weight management group

A combined dietary and exercise program will be used for childhood obesity treatment, as a systematic review suggests that combined dietary, physical activity, and behavioral components is effective in child and adolescent obesity treatment [20]. Furthermore, a previous study demonstrated the short- and long-term beneficial effects of a combined intervention program among obese children [29].

##### Dietary intervention

The participants and their parents will meet a dietitian six times during the 6 month program. Each family will be instructed to come to the first and last meetings with a completed 12 h dietary recall form [29,30]. The results will be evaluated using computer software and used to educate the participants and their parents on how to eat healthily. The meeting contents will include the reasons and risks of childhood obesity, healthy habits in food choices and food cooking, motivation for weight loss, food pyramid, food labels, eating habits, and recommended food sizes [29,30]. The worksheet and flyers on important nutritional issues will be distributed to the participants and their parents during these meetings. A trained nurse will telephone the parents of the participants in the intervention group once a week to offer support and encouragement [29].

##### Exercise intervention

The exercise intervention program will be adjusted on the basis of a previous study [31]. The participants and their parents will be asked to visit the Sport Medicine and Rehabilitation Center at Shanghai University of Sport once a week during the intervention period. The participants will be instructed to complete a 30 min aerobic exercise session on a treadmill or a bicycle during each exercise session. Each exercise session will be preceded by 10 min of warm-up, followed by 10 min of cool-down and stretching. The exercise intensity will be monitored using a heart rate monitor, and will be equivalent to a raise in heart rate from resting to 65% to 75% of the age-predicted maximal heart rate. An experienced exercise physiologist will be responsible for the training sessions. The participants will be instructed to complete two exercise sessions per week of the same exercise intensity and duration at home. The heart rate will be monitored by a heart rate monitor to ensure that the exercise intensity is consistent for each exercise session. The participants in the control group will be asked to keep their regular exercise habit during the first 6 months.

#### Control group

The participants in the control group will attend a 60 min group session per week. Each session will consist of a 30 min lecture, followed by a 30 min discussion. The lectures will cover health-related topics such as obesity, physical activity, and nutrition. The participants in the control group will be asked to maintain their previous lifestyle and not participate in any other regular exercise programs.

### Outcome measures

All outcome measure assessments will be administered by the main research assistant, who will not be informed of the group allocation, at 2 weeks (baseline), 6 months (follow-up 1, at the end of the intervention), and 12 months (follow-up 2). A demographic questionnaire will be completed at baseline. Demographic information includes the participants’ and their parents’ characteristics (that is, sex, age, ethnicity, family structure, postcode, and socioeconomic status).

#### Primary outcome measures

##### Anthropometric measurements

Body weight and height will be measured with each study participant in minimal clothing and bare feet. Body height will be measured to the nearest 0.5 cm using a fixed stadiometer (Holtain Ltd., Crymych, Dyfed, UK). Body weight will be measured to the nearest 0.1 kg using a standard scale (TBF 543 model; Tanita, Tokyo, Japan). Body mass index will be calculated as:

$$ \mathrm{Body}\ \mathrm{m}\mathrm{ass}\ \mathrm{in}\mathrm{dex}=\frac{\mathrm{Body}\ \mathrm{weight}\ \mathrm{in}\ \mathrm{kg}}{{\left(\mathrm{Body}\ \mathrm{height}\ \mathrm{in}\ \mathrm{m}\right)}^2} $$

Waist circumference will be recorded to the nearest 1 mm and measured at the midpoint between the lowest rib and the superior border of the iliac crest using an inelastic measuring tape [32]. Hip circumference will be measured at the maximum posterior extension of the buttocks [32].

##### Body fat percentage measurement

Dual-energy X-ray absorptiometry (GE Lunar Prodigy, software version 10.51.006, GE Healthcare, Madison, United States) will be used to measure body fat percentage. Each participant will be scanned in the supine position using X-rays at two energy sources (40 keV and 70 keV) in fast mode. A series of transverse scans will be performed from head to toe at 1 cm intervals. The time spent will be approximately 5 min, depending on the participant’s height.

#### Secondary outcome measures

##### Postural stability assessment

Postural control during daily living activities is regulated by the horizontal center of mass acceleration, which is mainly associated with the horizontal distance between the center of mass and center of pressure from a biomechanical perspective. The postural instability associated with obesity usually results in altered biomechanics strategies designed to improve the person’s sense of balance during locomotion [13]. Therefore, three-dimensional motion analysis will be used to measure kinematics and kinetics data, as well as to evaluate dynamic postural stability during different tasks.

Kinematics and kinetics data will be collected and analyzed using a Vicon Motion Analysis System (Vicon MX-13, Oxford Metrics, UK) with nine infrared cameras that record three-dimensional motion at 200 Hz and coupled with force plates (models 9286AA, Kistler Instruments Corp, Swaziland) that record ground reaction force at 1,000 Hz. The trajectories of 45 retro-reflective markers (14 mm in diameter) will be captured at different landmarks of the subject according to a Plug-in-Gait marker set.

These biomechanical data will be collected while each participant performs three tasks:

1. Level walking at normal speed, faster speed, and slow speed
  Normal cadence is the subject’s preferred speed. Fast speed is 30% faster than the normal walking speed, and slow speed is 10% slower than the normal walking speed [11]. Four force plates will be embedded on the ground in the middle of a walkway, so that the participants can plant their feet on each plate consecutively during walking.
2. Stair climbing
  The experimental staircase consists of six steps. The step dimensions are 17 cm (height) and 28 cm (tread) with a stair slope of 31° [33]. No handrail will be used. Three force platforms (9286AA, Kistler Instruments Corp, Switzerland) are embedded in the second, third, and fourth steps of the staircase. The participants will be asked to walk, ascend, and descend at their natural speed without any reference to the force platforms.
3. Rising from a seated position
  During the task, the participants will rise from a seated position from a 30 cm high bench. They will be instructed to sit on the edge of the bench, with each foot on a force plate equidistant from the center line, and to bend their knees so that their feet are slightly behind their knees [10].
  Static posture stability will be examined during the double-leg stance and tandem stance with eyes open and closed [11]. The subjects stand with two heels touching together and the feet externally rotated by 10° in the double-leg stance. The subjects stand with the dominant foot forward from heel to toe in the tandem stance. The center of pressure data will be collected from a force platform (9286AA, Kistler Instruments Corp, Switzerland) using BroWare data collection and analysis software with a sampling frequency of 1,000 Hz in a computer workstation. The subjects will be asked to stand as still and as quietly as possible with arms akimbo and barefoot to eliminate the effects of shoes, socks, and heel height. The required standing time is 30 s. If the participant loses balance in the period (steps to regain balance), he or she will be asked to repeat the test up to a maximum of three times. A 3 min rest will be allowed between two tests. The center of pressure displacement in the medial-lateral and anterior-posterior directions will be used to analyze postural stability.

##### Proprioception test of knee and ankle

This measurement method was reported in our previous studies [15]. Knee and ankle proprioception will be tested using an electrically driven movable frame. During the tests, the participant will sit on a chair with the dominant leg supported by the frame. The leg can be passively moved in a flexed or extended direction at a velocity of 0.4°/s. Once the participant is able to detect the leg motion, he or she will press a handheld stop button and confirm the direction of the motion. The rotation angles of the frame will be determined as the threshold for the detection of the knee and ankle joint. The mean values of the three trials in one direction will be used for the analysis.

##### Neuromuscular response

Neuromuscular response indicated by muscle latency will be assessed using electromyography (EMG) of the leg muscles while an unexpected perturbation is applied to the ankle. A customized trapdoor with an 18° tilt angle will be used to generate an ankle inversion perturbation while subjects stand barefoot on the trap doors. The Noraxon EMG system (Noraxon USA Inc., Scottsdale, USA) will be used to collect surface EMG signals from five muscles (rectus femoris, semitendinosus, gastrocnemius, peroneus longus, and anterior tibialis) of the right leg and onset signals at the trapdoor tilting with a sampling frequency of 1,000 Hz. Both feet will be randomly tilted at least seven times to decrease anticipatory effects. The onset latency of the muscles refers to the time interval in milliseconds (ms) between the trapdoor initiation and the first rising front of the EMG burst from the baseline. The EMG onset will be visually determined by an experienced researcher.

Furthermore, muscle activation will be determined during level walking and stair climbing. The EMG data will be collected from eight lower extremity muscles according to the recommended muscles for gait analysis from Winter and Yack [34]; these muscles are the ipsilateral and contralateral erector spinae at the level of the iliac crest, rectus femoris, vastus medialis, vastus medialis, tibialis anterior, biceps femoris, peroneus longus, and gastrocnemius. The Noraxon EMG system (Noraxon USA Inc., Scottsdale, USA) will be used to collect surface EMG signals from these muscles of the right leg with a sampling frequency of 1,000 Hz. The EMG signals from five complete gait cycles per task will be used in data reduction. Prior to the EMG data collection, maximal isometric contraction data will be gathered for each muscle. The mean EMG amplitude and on-off muscle timing during a gait cycle will be used in the data analysis [35].

##### Muscle strength test of knee and ankle

**Figure 1:**
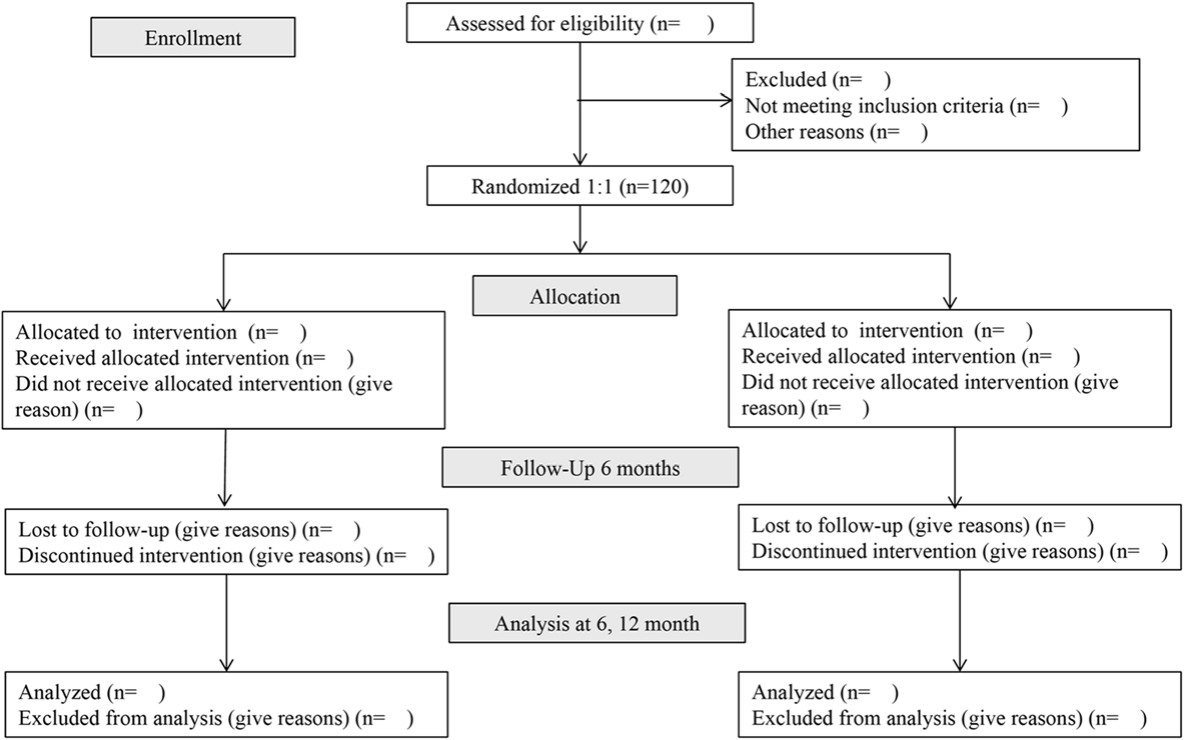
**Flow diagram of study design.**

Muscle strength of the dominant knee and ankle joint will be tested using an isokinetic dynamometer [17]. Participants will perform three maximum concentric contractions for the knee extensors and flexors at an angular velocity of 60°/s. The highest peak torque that indicates muscle strength will be normalized by kilogram of body weight. The dynamic endurance of the knee extensors and flexors will be assessed by measuring 40 repeated maximum isokinetic contractions at an angular velocity of 180°/s. The work in moving from a knee angle of 80° to a knee angle of 10° will be recorded for each contraction. The endurance index is defined as the ratio of the work performed during the last five contractions over the first five contractions. Ankle dorsiflexor and plantarflexor strength will be measured at an angular velocity of 60°/s. Participants will be instructed to push a foot away from themselves and then pull it toward themselves at the maximum velocity for each action. Peak torque will be determined as the highest torque generated from the three trials. Relative peak torque will also be analyzed (Figure 1).

### Statistical analysis

Statistical analysis will be performed using SPSS for Windows version 17.0 (SPSS Inc. Chicago, IL). Data will be presented as the mean and standard deviation in which the distribution of variables is normal. Potential covariates will be determined at the baseline (that is, weight status, growth potential, sex, age, and socioeconomic status), and the difference by study groups or sex will be determined using one- or two-way analysis of variance or *χ*^2^ test. Baseline differences between those who attend and those who do not attend the follow-up measurements will be examined using separate variance *t* tests.

Intention-to-treat analysis will be performed by including all participants in the analysis according to the original group allocation. The follow-up will be maximized regardless of program attendance. A linear mixed model, which includes time (as a repeated factor), group, their interaction, and Bonferroni correction for *post-hoc* multiple comparisons, will be used to determine whether a significant time by group effect exists at the baseline, and after 6 months and 12 months. Average intervention effects of the follow-up period will be estimated and tested using the Bonferroni method for *post-hoc* analysis, in which groups by time interactions are insignificant.

## Discussion

This study attempts to examine the effect of a 6 month combined physical activity and nutritional intervention on postural stability and neuromuscular function among obese children. Previous studies found that physical exercise and a nutritional guidance program have beneficial effects on the anthropometric parameters and cardiovascular risk factors [36,37]. Furthermore, weight reduction in obese children is associated with increased physical fitness and improved movement skills [20,21]. As previously mentioned, maintaining a stable posture is essential for many daily activities and injury prevention. Obese children have poorer postural stability than their normal-weight counterparts, and consequently experience greater difficulty in daily physical activities, leading to a higher risk of falling and fractures [25,26]. However, only a few studies have examined the effect of weight management interventions on postural stability and discussed the possible reasons for this in obese children. Postural stability and control are related to the functions of vision, vestibular, proprioception, and neuromuscular control. The larger body size, body mass, abnormal body fat distribution, and potential neuromuscular disadvantage of obese children during this period can contribute to postural instability [13]. Previous studies have found that several sensory receptors in obese individuals could be associated with postural instability [11,15]. Findings from a recent study show that a 6 month weight loss program for obese children is associated with improved postural stability and decreased potential vestibular stress or disturbances [24]. Moreover, findings from obese adults show that a weight loss program improves static postural stability; the decrease in plantar contact area enables mechanoreceptors to better detect postural oscillations [22]. As previously mentioned, postural instability in the obese population may be related to larger anthropometric parameters, abnormal body fat distribution, and poor neuromuscular control. To our knowledge, no study has investigated the effects of a weight management program on neuromuscular function among obese children. Therefore, the findings of this study will fill the research gap in question. Furthermore, the possible mechanism of postural instability in obese children may be discussed.

The strengths of our protocol are as follows: (1) the use of combined physical activity and nutritional intervention in postural stability and neuromuscular function among obese children, which has not been previously described in this population; (2) the relatively long study duration, with an intervention period of 6 months and a follow-up period (with no active intervention) of 6 months, for a total study period of 12 months; (3) extensive follow-up to monitor the effects of the combined physical activity and nutritional intervention on postural stability and neuromuscular function among obese children; (4) evaluation of dynamic and static postural stability using fully three-dimensional biomechanical analysis to determine dynamic postural stability and provide a quantitative analysis of the intervention effect; (5) measurement of the neuromuscular function to provide advanced findings to explain the possible mechanisms of postural instability in obese children; and (6) a comprehensive dissemination plan to ensure the adequate uptake of knowledge generated in this study. However, this study also has several limitations. First, recruitment is limited to prepubescent obese children, and thus the study results may only be valid for prepubescent obese children. The use of a larger sample size will also address the current study’s limitation of relying on a relatively small study population and the fact that it is not a multicenter trial. Moreover, we will not compare the efficacy of the weight loss program with that of other weight loss programs because of the limited subject number.

In conclusion, this study attempts to estimate the effect of weight loss intervention on outcomes, including daily life function, postural stability, and neuromuscular control in prepubescent obese children. The study results may provide evidence to support the beneficial effects of a weight loss management program on postural stability and neuromuscular control, potential vestibular stress or disturbances, and a falling probability of obese children. Further comprehensive research on the weight management program for obese population will be proposed on the basis of the proposed project.

### Trial status

Participant recruitment.

## Abbreviation

EMG: Electromyography
